# The Role of *TP53* in Cisplatin Resistance in Mediastinal and Testicular Germ Cell Tumors

**DOI:** 10.3390/ijms222111774

**Published:** 2021-10-29

**Authors:** Dennis M. Timmerman, Thomas F. Eleveld, Ad J. M. Gillis, Carlijn C. Friedrichs, Sanne Hillenius, Tessa L. Remmers, Sruthi Sriram, Leendert H. J. Looijenga

**Affiliations:** Princess Máxima Center for Pediatric Oncology, Heidelberglaan 25, 3584 CS Utrecht, The Netherlands; D.M.Timmerman-6@prinsesmaximacentrum.nl (D.M.T.); t.f.eleveld@prinsesmaximacentrum.nl (T.F.E.); A.J.M.Gillis@prinsesmaximacentrum.nl (A.J.M.G.); c.c.friedrichs-2@prinsesmaximacentrum.nl (C.C.F.); hillenius.sanne@gmail.com (S.H.); tessa.l.remmers@gmail.com (T.L.R.); s.s.sriram@prinsesmaximacentrum.nl (S.S.)

**Keywords:** human malignant germ cell tumors, mediastinal germ cell tumors, testicular germ cell tumors, cisplatin resistance, *TP53*, NCCIT, 2102Ep, stratification

## Abstract

Germ cell tumors (GCTs) are considered to be highly curable; however, there are major differences in the outcomes related to histology and anatomical localization. GCTs originating from the testis are, overall, sensitive to platinum-based chemotherapy, whereas GCTs originating from the mediastinum show a worse response, which remains largely unexplained. Here, we address the differences among GCTs from two different anatomical locations (testicular versus mediastinal/extragonadal), with a specific focus on the role of the P53 pathway. It was recently shown that GCTs with *TP53* mutations most often localize to the mediastinum. To elucidate the underlying mechanism, *TP53* knock-out lines were generated in cisplatin-sensitive and -resistant clones of the representative 2102Ep cell line (wild-type *TP53* testicular GCT) and NCCIT cell line (hemizygously mutated *TP53*, mutant *TP53* mediastinal GCT). The full knock-out of *TP53* in 2102Ep and resistant NCCIT resulted in an increase in cisplatin resistance, suggesting a contributing role for P53, even in NCCIT, in which P53 had been reported to be non-functional. In conclusion, these results suggest that *TP53* mutations contribute to the cisplatin-resistant phenotype of mediastinal GCTs and, therefore, are a potential candidate for targeted treatment. This knowledge provides a novel model system to elucidate the underlying mechanism of clinical behavior and possible alternative treatment of the *TP53* mutant and mediastinal GCTs.

## 1. Introduction

Germ cell tumors (GCTs) are the most common solid malignancies in young men [1,2]. Despite the high frequency of these cancers within this defined age group, the discovery of the exceptional sensitivity of these tumors to the platinum DNA crosslinking compound cisplatin has led to the survival of most patients, with the current five-year survival rate exceeding 95% [3,4,5]. As GCTs are derived from embryonic germ cells, closely resembling embryonic stem cells, their hypersensitivity to DNA-damaging agents is often traced back to their early embryonic phenotype [6,7,8]; for instance, similarly to embryonic stem cells, GCTs often display a low/inefficient DNA damage response and, as opposed to most solid malignancies, GCTs that are naïve to systemic treatment rarely harbor *TP53* mutations, irrespective of histology [9,10]. Moreover, the wild-type *TP53* status of GCTs, combined with a pluripotent phenotype, high levels of PUMA and NOXA, and, often, low expression levels of *CDKN1A* (P21), result in a cellular disbalance and a favor towards apoptosis over DNA repair [11,12,13,14,15]. Furthermore, a physiological antagonist of P53, mouse double minute 2 homologue (MDM2), has been illustrated to be especially important in P53 regulation in GCTs, as it has been shown to hamper the apoptotic response via binding to P53 and can be a putative important clinical target [8,16,17,18,19]. It has already been shown that the inhibition of MDM2 and disruption of the MDM2–P53 interaction can potentiate apoptosis and sensitize GCT cells to cisplatin [16,17]. On the other hand, no correlation has been identified between the levels of MDM2 and the treatment response [10]. Furthermore, the existence of many MDM2 binding partners, and the reported synergy between MDM2 antagonists and (targeted) therapy, both in GCTs and other cancers, make this an interesting and relevant target as well [16,17,20,21]. Histologically and clinically, GCTs can be divided into two main subtypes, referring partly to their pluripotent potential, namely, seminomas and non-seminomas [6,7]. While patients presenting with seminomas have an excellent prognosis, patients harboring non-seminomas have a mixed prognosis, based on tumor histology (e.g., embryonal carcinoma (EC), yolk sac tumor (YST), choriocarcinoma (CC), or teratoma (TE)), therapy naivety or chemotherapeutic resistance, and anatomical location, mainly focusing on extra-cranial GCTs of the mediastinum versus the testis [6,7,9,14,22]. Apart from tumor histology and origin, the P53 pathway and deregulation thereof has been studied in light of GCT treatment resistance [8,9,10,13,14,16,17,19,23]. Even though P53′s have many implications in resistance, no clear-cut result has been obtained that displays their role in clinical resistance, especially related to informative in vitro models [10,23]. In this study, we focused on the latter (i.e., mediastinal GCTs vs. testicular GCTs) and developed a novel approach to shed light on the difference in treatment resistance between testicular and mediastinal GCTs. This is an important issue, as it is currently unclear whether mediastinal GCTs are more resistant to treatment because of their *TP53* mutations, or whether these mutations simply occur more in these tumors as these tumors harbor different intrinsic resistance mechanisms. To this end, we wanted to elucidate whether the removal of *TP53* in a testicular GCT cell line can convey cisplatin resistance, and, thus, (partly) explain mediastinal GCT aggressiveness and treatment resistance. Firstly, we used the online cBioPortal tool to analyze an extensive GCT patient data set containing detailed clinical information, including treatment, tumor resistance, tumor stage, anatomical location, histology, and genetic mutations [9]. Furthermore, two well-established GCT cell lines, originating from different anatomical locations and harboring a different *TP53* background (*TP53* mutant/loss in NCCIT from the mediastinum and *TP53* wild-type in 2102Ep from the testis), were modified using a CRISPR/Cas9-mediated *TP53* knock-out model system. We subsequently investigated the difference in cisplatin resistance in these testicular and mediastinal GCT cell lines. Using both GCT patient data characteristics and functional mechanistic cell line investigations, we show a role of P53 in GCT cisplatin resistance related to the anatomical location of the tumor.

## 2. Results

### 2.1. Presence of TP53 Mutations in Refractory Cisplatin-Resistant GCTs with a Specificity towards Mediastinal Localization

**Figure 1:**
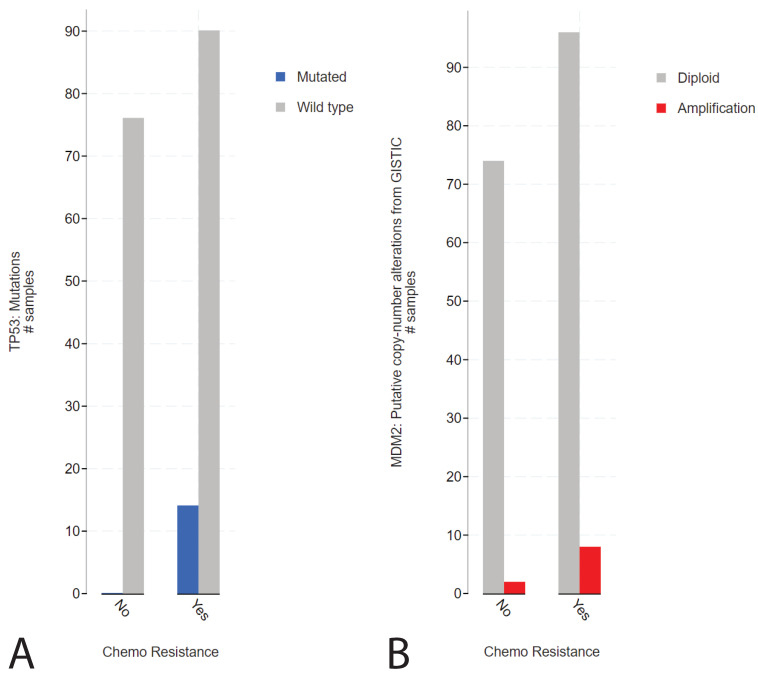
cBioPortal analysis of the tumor resistance in *TP53*- or *MDM2*-altered patients in the MSKCC, J Clin Oncol 2016 data set. (**A**) Bar graph displaying the number of patients with sensitive or resistant cisplatin, patients harboring wild-type (grey) or mutated (blue) *TP53* are plotted. (**B**) Bar graph displaying the number of patients with sensitive or resistant cisplatin, patients harboring wild-type (grey) or amplified (red) *MDM2* are plotted [9,24,25].

To elucidate the function of P53 in (resistant) GCTs, we initially used the cBioPortal online tool. We investigated the MSKCC data set on refractory GCTs previously reported by Bagrodia and colleagues in 2016 [9]. The rationale for investigating this data set was based on the abundant presence of detailed clinical data, including anatomical location, treatment, number of chemotherapy cycles, patient survival and outcome, and tumor histology. Supplemental Figure S1A illustrates the presence of *TP53* (and *MDM2*) alterations in cisplatin-sensitive and -resistant GCTs. Strikingly, while GCTs rarely harbor *TP53* mutations, in line with their embryonic phenotype [8], alterations in *TP53* are detected in cisplatin-resistant patients. Furthermore, we observe that *MDM2* amplifications become increasingly abundant in patients with cisplatin-resistant GCTs. Note that, as expected, alterations regarding *TP53* are often missense mutations or deep deletions. When comparing the disease-free survival of patients with alterations in the *TP53* gene to patients with wild-type *TP53* (unaltered group), we observed a highly significant (logrank test *p*-value of 1.991 × 10^−6^) decrease in disease-free survival in patients harboring *TP53* alterations (Supplemental Figure S1B). As previously reported, there could be a bias in this analysis, associated with the type of genetic aberration in relation to the anatomic location of GCTs [9]. The tumors of patients harboring *TP53* mutations often localize to the mediastinum, whereas the tumors of patients harboring *MDM2* amplifications primarily localize to the testis (Supplemental Figure S1C,D). Interestingly, *TP53* or *MDM2* aberrations occur significantly more frequently in patients with chemotherapy-resistant tumors (Figure 1A,B).

### 2.2. Mediastinal GCT Cell Line NCCIT Harbors Low Levels of MDM2 and Mutant TP53 whereas Testicular GCT Cell Line 2102Ep Harbors Wild-Type TP53 and High Levels of MDM2

**Figure 2:**
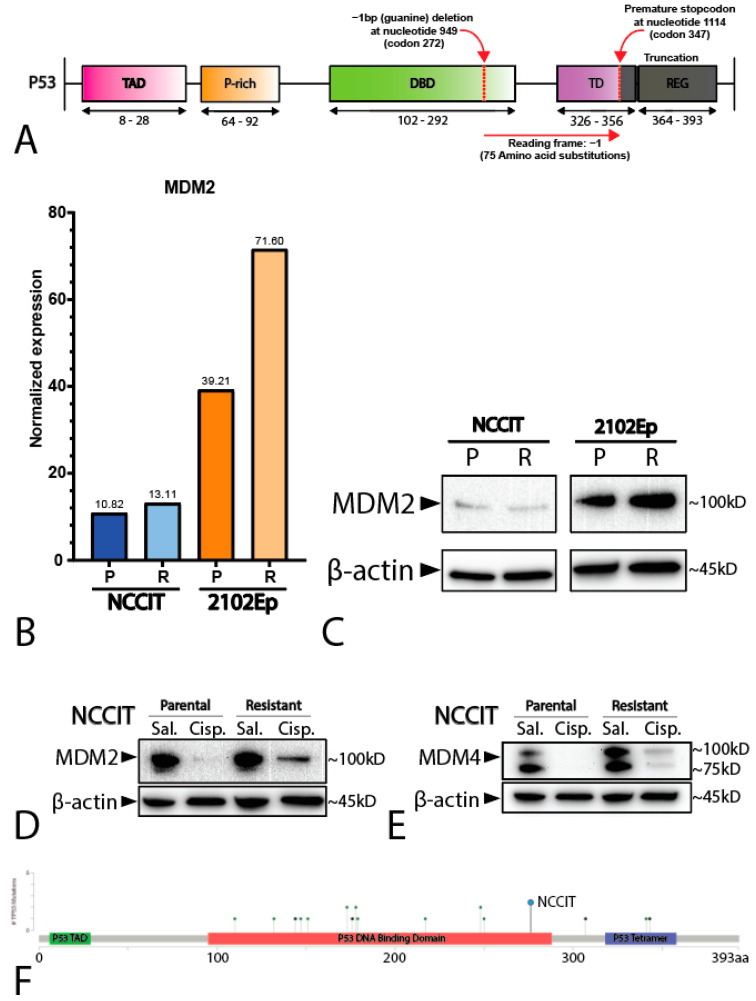
Characterization of the cell lines NCCIT and 2102Ep. (**A**) Schematic overview of the hemizygous mutation present in the NCCIT cell line. (**B**) Bar graph displaying the normalized expression (RNA-seq) of *MDM2* in the NCCIT and 2102Ep parental and resistant cell lines. (**C**) Western blot showing the protein levels of MDM2 in the NCCIT and 2102Ep parental and resistant cell lines. (**D**,**E**) Western blot displaying the MDM2 (**D**) and MDM4 (**E**) protein levels after treatment with sublethal cisplatin doses (1 µM) or saline vehicle control. (**F**) Mutational position of *TP53* mutations in patients in the MSKCC, J Clin Oncol 2016 data set. The mutation found in NCCIT is highlighted with a blue dot.

To study the difference between mediastinal and testicular GCTs, we used the well-established and -characterized NCCIT and 2102Ep GCT (EC) cell lines. While 2102Ep originates from the testis, NCCIT originates from the mediastinum, with a similar differentiation state [26]. Furthermore, similarly to most GCTs, 2120Ep has a wild-type *TP53* status, whereas NCCIT carries a hemizygous one-base-pair deletion at nucleotide 949 (codon 272), resulting in a frameshift and a premature STOP codon at codon 347 (Figure 2A). This observation is in line with the finding of *TP53* mutations in mediastinal GCTs (see above).

Additionally, we employed matched isogenic clones of both NCCIT and 2102Ep that have acquired a cisplatin resistance phenotype through long-term sublethal exposure to cisplatin (see Materials and Methods section for details). RNA sequencing (RNA-seq) analysis showed that both the parental and resistant NCCIT cell lines had lower normalized *MDM2* expression than both the 2102Ep cell lines, with 2102Ep resistance displaying the highest levels of *MDM2* (Figure 2B), supported by Western blotting showing that the resistant 2102Ep subclone had higher levels of MDM2 (Figure 2C). In contrast, the expression levels of *MDM4* were similar between all the cell lines (Supplemental Figure S2). Principal component analysis of the matched parental and resistant cell lines showed no major differences and demonstrated close similarities between the matched subclones (data not shown). To determine whether NCCIT had an active DNA damage response and possible P53 pathway activation, despite a low MDM2 level, we treated NCCIT cells with sublethal (1 µM) levels of cisplatin for 24 h prior to protein analysis via Western blotting. Both the NCCIT parental and resistant cell lines showed a clear decrease in MDM2 and MDM4 after exposure to cisplatin, an effect that was not visible in the saline vehicle control condition (Figure 2D,E). This indicates a functional DNA-damage sensing pathway upstream of MDM2 and MDM4, and, therefore, suggests an intact regulation of P53 downstream of MDM2 and MDM4, despite the suggested null status of *TP53* as described in the literature [13,16,23,27].

### 2.3. P53 Is Involved in Cisplatin Resistance in Both Wild-Type (Testicular) and Mutant (Mediastinal) GCT Cell Lines

**Figure 3:**
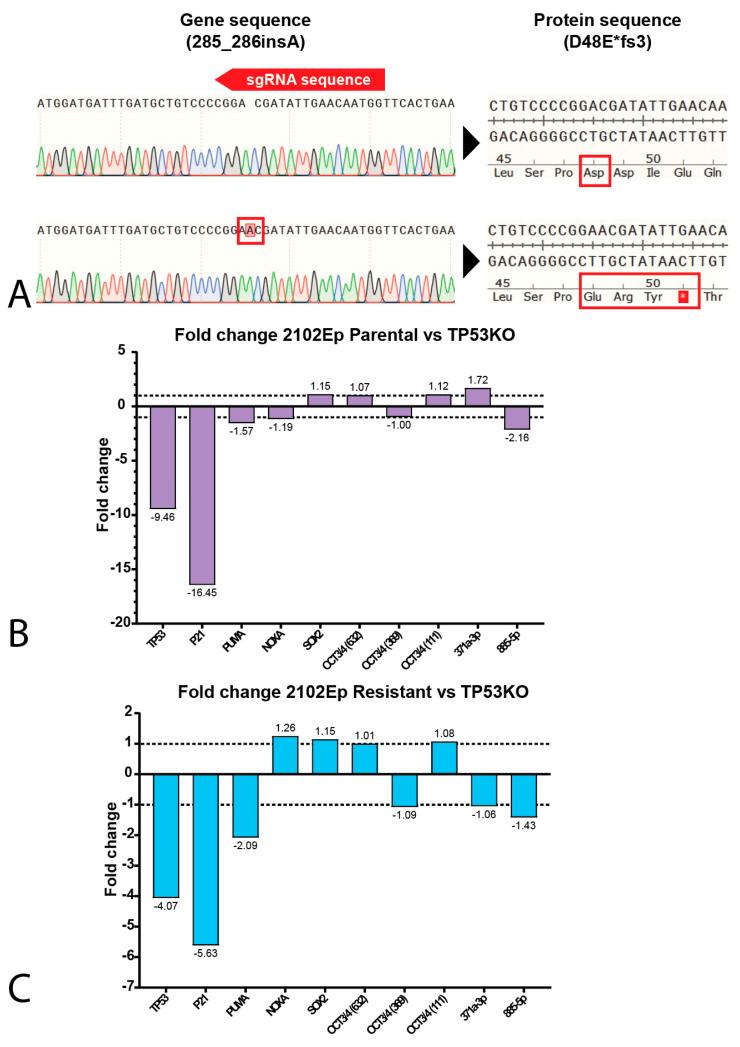

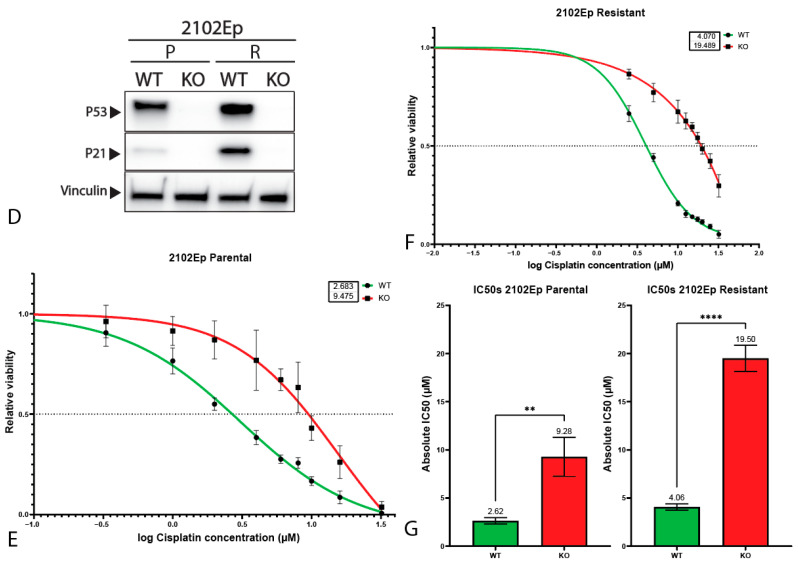
Characterization of 2102Ep *TP53* knock-out cell lines. (**A**) SnapGene genome sequence alignments of the CRISPR/Cas9 target site of the *TP53* gene. The knock-out cell line (bottom sequence) shows a one-base-pair insertion (**A**) at amino acid 49, resulting in a premature STOP codon at amino acid 51. (**B**,**C**) Bar graphs showing the fold change in expression between 2102Ep parental cell line and its isogenic *TP53* knock-out clone (**B**) or 2102Ep-resistant cell line and its isogenic *TP53* knock-out clone (**C**). (**D**) Western blots showing the protein levels of P53, P21 and vinculin (as loading control) in 2102Ep parental and resistant cell lines and their isogenic *TP53* knock-out clones. (**E**,**F**) S-curves showing the viability of the parental (**E**) and resistant (**F**) 2102Ep cell lines and their corresponding knock-out when treated with cisplatin for 72 h. Graphs represent three biological replicates with three technical replicates each. (**G**) Bar plots displaying IC50 values of all 2102Ep cell lines. Both cell line pairs show significant differences in IC50 values after knock-out (parental *p* = 0.0049, resistant *p* ≤ 0.0001, unpaired Student’s *t*-test). Mean ± SD: 2102Ep parental 2.62 ± 0.33, 2102Ep parental *TP53* KO 9.28 ± 2.02, 2102Ep resistant 4.06 ± 0.32, and 2102Ep resistant *TP53* KO 19.50 ± 1.36. Graphs represent three biological replicates with three technical replicates each. ** *p* ≤ 0.01, **** *p* ≤ 0.0001.

**Figure 4:**
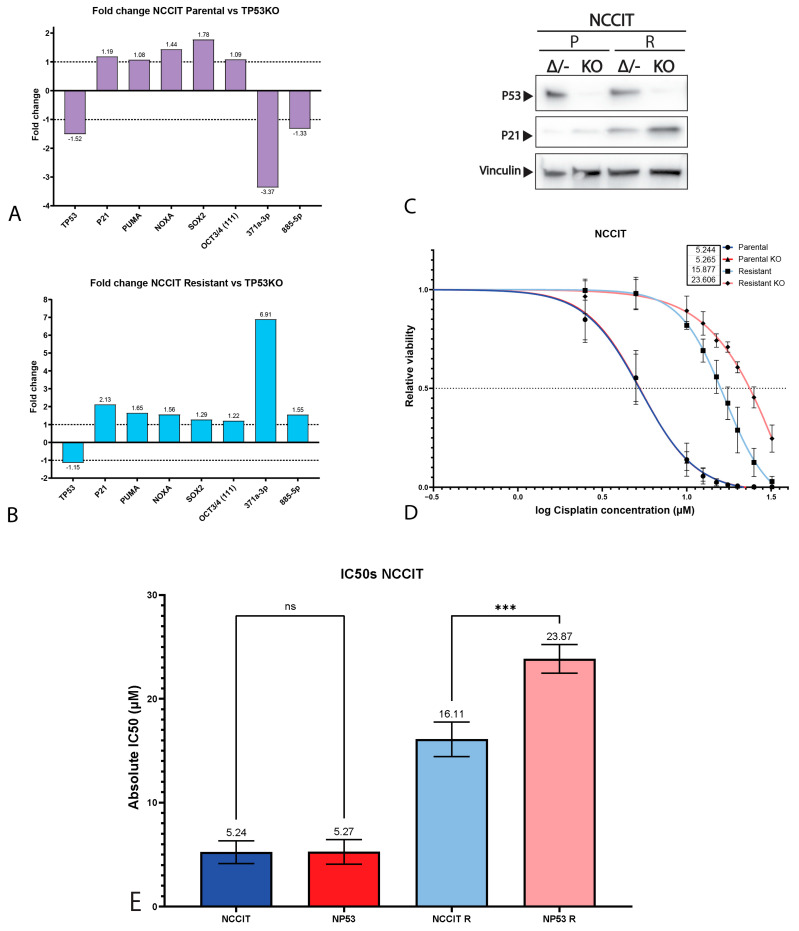
Characterization of NCCIT *TP53* knock-out cell lines. (**A**,**B**) Bar graphs showing the fold change in expression between NCCIT parental cell line and its isogenic *TP53* knock-out clone (**A**) or NCCIT-resistant cell line and its isogenic *TP53* knock-out clone (**B**). (**C**) Western blots showing the protein levels of P53, P21 and vinculin (as loading control) in NCCIT parental and resistant cell lines and their isogenic *TP53* knock-out clones. (**D**) S-curves showing the viability of the NCCIT cell lines (parental and resistant and *TP53* knock-out lines) when treated with cisplatin for 72 h. Graphs represent three biological replicates with three techinical replicates each. (**E**) Bar plots displaying IC50 values of all NCCIT cell lines. The NCCIT-resistant cell line shows a significant difference in IC50 values after knock-out (*p* = 0.0005, one-way ANOVA, Tukey’s multiple comparisons post hoc test). Mean ± SD: NCCIT parental 5.24 ± 1.09, NCCIT parental *TP53* KO 5.27 ± 1.18, NCCIT resistant 16.11 ± 1.67, and NCCIT resistant *TP53* KO 23.87 ± 1.38. ns = *p* > 0.05, *** *p* ≤ 0.001.

It is largely accepted that the chemotherapeutic hypersensitivity of GCTs is partly due to their wild-type *TP53* status [8,14,28,29,30]. However, despite its *TP53* mutant status, the NCCIT cell line is considered to be inherently sensitive to cisplatin. Thus, we further compared the mutational status of the NCCIT cell line to the mutations found in refractory GCT patients (Figure 2F). When comparing the intrinsic *TP53* mutation in the NCCIT cell line to the *TP53* mutations present in refractory GCT patients, we observed that most mutations found in patients disrupt the DNA-binding domain of *TP53*, a well-known mutational hotspot [9,31]. In contrast, the intrinsic *TP53* mutation of NCCIT appears to largely spare the DNA-binding domain and is, therefore, more C-terminally located than most mutations found in refractory patients, suggesting the possibility for residual protein activity. Furthermore, the enrichment of mutations in *TP53* in refractory patients, together with a bias towards mediastinal anatomical localization (and, hence, a more resistant phenotype), suggests that *TP53* mutations could add additively to inherent cisplatin resistance mechanisms [9,14,22]. Based on these observations, we decided to further test the involvement of *TP53* in cisplatin resistance in the approach described. Therefore, we generated isogenic CRISPR/Cas9-mediated *TP53* knock-out clones of both 2102Ep and NCCIT, as well as their resistant counterparts. Sanger sequencing, after mono-clonal picking and expansion, revealed mono-clonal sequence traces and a one-base-pair insertion at amino acid 48, resulting in a premature STOP codon at amino acid 51 (Figure 3A). We were able to obtain clones harboring this mutation for all the investigated cell lines. No major copy number changes between the original and *TP53* knock-out NCCIT and 2102Ep subclones were identified based on Infinium Global Screening Array-24 v3.0 BeadChipGSA (GSA) profiling (Supplemental Figure S3). Gene expression analysis using RT-qPCR indicated a clear reduction in both *TP53* and *CDKN1A* (P21) expression in both 2102Ep parental *TP53* knock-out lines (~9.46 and ~16.45, respectively) and 2102Ep-resistant *TP53* knock-out lines (~4.07 and 5.63, respectively), which was also confirmed by Western blot (Figure 3B–D). No differences were observed in P53 target gene expression (PUMA/NOXA) or differentiation marker expression (SOX2, OCT3/4, miR371a-3p, or miR885-5p); the latter indicates that the loss of *TP53* expression had no effect on the differentiation status. The miR371a-3p expression levels were checked because of its many implications in GCTs (mostly as a biomarker and marker of pluripotency in these tumors), together with the implications of P53 pathway regulation [7,20,32,33,34,35,36,37,38]. Strikingly, after treating 2102Ep parental and resistant cells, as well as their isogenic *TP53* knock-out clones, with cisplatin, we identified a clear significant (parental *p* = 0.0049, resistant *p* ≤ 0.0001) shift in cisplatin resistance when comparing the *TP53* knock-out clone to its wild-type counterpart, with the 2102Ep-resistant *TP53* knock-out clone demonstrating the highest cisplatin resistance (Figure 3E–G). When we performed this approach with the NCCIT cell line, we obtained clones with the same one-base-pair insertion mutation (A) found in the 2102Ep cell lines (Figure 3A). However, we found no strong reduction in either *TP53* or *CDKN1A* expression, P53 target gene expression, or differentiation marker expression (Figure 4A,B). Interestingly, however, we did observe a reduction in miR371a-3p expression (3.37-fold) in the parental *TP53* knock-out clone compared to its parental counterpart, while we observed an increase in miR371a-3p expression (6.91-fold) in the NCCIT-resistant *TP53* knock-out clone compared to its NCCIT-resistant counterpart (Figure 4A,B). Western blotting confirmed that the *TP53* knock-out lines had lost P53 protein expression; however, strikingly, the levels of P21 were increased in the NCCIT-resistant *TP53* knock-out line compared to its NCCIT-resistant counterpart (and both other lines; Figure 4C). Moreover, *TP53* knock-out in the NCCIT clones resulted in no shift in cisplatin resistance in the NCCIT parental clone, and a major significant (*p* = 0.0005) shift in cisplatin resistance in the NCCIT-resistant *TP53* knock-out clone compared to its NCCIT-resistant counterpart (Figure 4D,E).

## 3. Discussion

Here, we present a study on the effect of the loss of *TP53* in two well-studied GCT cell lines, representative of a mediastinal and testicular origin, as a starting point to further investigate the role of *TP53* in cisplatin resistance in GCTs, in relation to their anatomical localization. Based on the initial analysis of refractory GCT patients, we observed an overrepresentation of *TP53* mutations and *MDM2* amplification in proven cisplatin-resistant tumors [9]. Of note, only one patient harbored a *MDM4* alteration (missense mutation of unknown significance) [9]. This low frequency of *MDM4* alterations is not fully unexpected, as MDM4 is not able to directly ubiquitinate P53 and target it for proteasomal degradation; this is in stark contrast to MDM2, which is able to directly downregulate P53 through proteasomal degradation [8,21,39]. Subsequent analysis showed that tumors harboring *MDM2* amplifications were mostly testicular in origin, whereas *TP53*-mutated tumors were primarily mediastinal [9]. As reported previously and as part of the International Germ Cell Cancer Collaborative Group (IGCCCG) risk stratification, patients harboring a primary mediastinal GCT have the worst prognosis [40]. One could speculate that the bias for *TP53* mutations in mediastinal GCTs could be due to a less favorable niche and more strict selection for these tumors [41]. As mentioned before, the embryonal origin of these tumors is still in favor of genome protection and an intact *TP53* signaling pathway [8]. It could well be that mediastinal GCTs are on the cross-roads between unfavorable niche selection and, thus, a bias towards *TP53* mutations, and, thereby, a more treatment-resistant phenotype. In this study, we tried to elucidate if there was a causal link between the poor prognosis for mediastinal GCTs and their bias towards *TP53* mutations. In line with cBioPortal analysis, both the Western blot and RNA-seq data indicated higher MDM2 protein levels and a higher *MDM2* expression in the testicular 2102Ep GCT cell line compared to the mediastinal NCCIT GCT cell line, despite the lack of *MDM2* amplifications in 2102Ep. This suggests a mechanism for treatment resistance within wild-type *TP53* GCTs via MDM2 [16]. This is even more interesting in light of the many MDM2 interacting proteins and implications for MDM2 antagonists as anti-cancer therapies, working synergistically with both chemotherapeutics and targeted therapies [16,21,42]. We demonstrate that there are no differences in the RNA expression of *MDM4* between the mediastinal and testicular cell lines. Finally, as P53 is known to regulate MDM2 and MDM4, and could, therefore, interfere with DNA-damage sensing, we studied the upstream activity of the DNA-damage signaling pathway in the *TP53*-mutated NCCIT cell line [8,27]. Cells were treated with a sublethal dose of cisplatin (1 µM), and we observed a strong reduction in both MDM2 and MDM4 levels in both the parental and resistant cell lines. This indicates a functioning DNA-damage sensing response in the NCCIT cell line [27,43,44,45]. Of note, we identified a stronger reduction in MDM2 and MDM4 after cisplatin treatment in the parental cell line, most likely related to the already higher cisplatin resistance in the NCCIT-resistant cell line that functions upstream of the DNA-damage sensing response [27,43,44,45]. When comparing the *TP53* mutation present in the NCCIT cell line to the mutational profile of *TP53* mutations in refractory GCT patients, we noticed that the NCCIT mutant was located more distally within the protein and was unlikely to fully disrupt the function of the DNA-binding domain [9]. To test the function of *TP53* in GCT cisplatin resistance and to interrogate the functionality of the mutant *TP53* in the NCCIT cell line, we generated CRISPR/Cas9-mediated *TP53* knock-out cell lines. As the loss of *TP53* can contribute to chromosomal instability and, therefore, cisplatin resistance, we used GSA analysis to verify the genomic changes between the original and knock-out clones. No major copy number changes were identified related to the *TP53* knock-out procedure and subsequent selection. However, small copy number alterations could be observed, possibly related to clonal selection and expansion. Interestingly, although rarely observed in testicular GCT patients, full knock-out of *TP53* in the testicular EC cell line 2102Ep resulted in a significant increase in cisplatin resistance in both a parental and resistant background. This coincided with both the reduced expression levels of *TP53* and *CDKN1A* (P21), as well as the reduced protein levels of P53 and P21, suggesting a strong dependency on intact *TP53* for *CDKN1A* expression in this cell line. Strikingly, when we knocked out *TP53* in the *TP53* mutant mediastinal EC cell line NCCIT, we also observed an increase in cisplatin resistance in the resistant cell line only. The knock-out of *TP53* resulted in a full loss of P53 at the protein level and a minor reduction at the mRNA level. Interestingly, the parental *TP53* knock-out cell line showed reduced expression of microRNA371a-3p, while the resistant *TP53* knock-out line showed an increase. It remains to be elucidated whether this NCCIT observation is a passenger effect or is possibly related to previous findings regarding the negative regulatory effect of this microRNA cluster on *TP53* expression [32]. The absence of a lack of expression in the case of *TP53* knock-out is relevant in the context of the informativity of this molecular biomarker for GCTs, as recently reviewed by Leão and colleagues, as well as in the case of refractory disease [38]. In contrast, no effect on differentiation status was identified, demonstrating that the knock-out of *TP53* does not induce differentiation, excluding the possibility of increased resistance due to a differentiated phenotype [46]. It is important to note that despite the *TP53* status of these cell lines, being wild-type, hemizygous mutant, or full knock-out, it did not interfere with the detection of the microRNA cluster 371a-3. In other words, the miR371a-3 cluster appears to retain its suitability as a GCT biomarker irrespective of the tumor’s *TP53* status and, thus, also partially the level of cisplatin resistance [20,32,33,34,35,36,37,38,47,48,49]. The knock-out of *TP53* in the NCCIT-resistant (but not 2102Ep) cell line resulted in increased protein levels of P21, an effect not readily observed at the mRNA level, suggesting a negative effect on the P21 levels of the mutant *TP53* present in the resistant NCCIT subclone. This is interesting in light of previous studies indicating increased tumor resistance, malignancy, and aggressiveness caused by the P53-independent upregulation of P21 in *TP53* mutant tumors [50,51,52,53,54].

## 4. Materials and Methods

### 4.1. Cell Culture

The parental (T)GCT cell lines used were previously reported and further characterized by us [26]. NCCIT (RRID:CVCL_1451) was cultured in Roswell Park Memorial Institute (RPMI) 1640 medium with GlutamaxTM-I (Gibco; Thermo Fischer Scientific, Bleiswijk, The Netherlands) and 2102Ep (RRID:CVCL_C522) was cultured in Dulbecco’s modified Eagle’s medium (DMEM) with 4.5 g/L D-glucose with L-glutamine (Gibco; Thermo Fischer Scientific, Bleiswijk, The Netherlands) [55,56]. Media were supplemented with 10% fetal bovine serum and 1% penicillin–streptomycin (Gibco; Thermo Fischer Scientific, Bleiswijk, The Netherlands). The cell lines were cultured at 37 °C and 5% CO_2_. The resistant isogenic clones were generated by the group of Christoph Oing and Friedemann Honecker, University of Hamburg, Germany. These resistant cell lines were obtained by exposing the parental cell lines to cisplatin over a period of 6–9 months to increase the sublethal concentrations of these cells to cisplatin. Cisplatin dose was kept the same for two subsequent treatments and stepwise increased by 30–50%. Each cisplatin treatment was applied for 24 h in 80% confluent cells, followed by a medium change. Cells were allowed to rest with regular medium change over a period of 5–7 days until re-growth was detectable, and cells were re-plated afterwards. After one passage to regenerate, the next treatment was applied as mentioned above. Cells were passaged for a maximum of 7 cycles, followed by intermittent cryopreservation to prevent differentiation. For experimental use, obtained resistant subclones were cultured similarly to their sensitive parental lines without continuous cisplatin supplementation. The resistant subclones were maintained similarly to the parental cells (i.e., without cisplatin).

### 4.2. cBioPortal

Patient data were analyzed by using cBioPortal [24,25] by visiting cBioportal for Cancer Genomics. Available online: https://www.cbioportal.org/ (accessed on 28 September 2021) and selecting the germ cell tumors (MSKCC, J. Clin. Oncol. 2016) data set under testis tumors [9]. We queried for *TP53* and *MDM2* in the gene query section. Subsequent analysis was performed using the tools provided by cBioPortal.

### 4.3. RNA-seq

STAR (v2.5.3a) was used as aligner for RNA-seq data, using 2-pass mapping for each sample separately. Mapping quality plots were generated and checked based on sambamba flagstat (v0.6.7) statistics. Count files, with the number of RNA-seq reads for each gene were created with subread FeatureCounts (v1.5.2) and normalized for library size to counts per million (CPM).

### 4.4. Western Blot

Cell lysates were made using RIPA buffer, followed by measuring protein concentrations according to Pierce BCA Protein Assay Kit (Gibco; Thermo Fischer Scientific, Bleiswijk, The Netherlands). A total of 25 µg protein was loaded onto a 4–15% Mini-Protean TGX Stain-Free Protein Gel (Bio-Rad Laboratories, Lunteren, The Netherlands). After separation, the proteins were transferred to a 0.2 µm PVDF membrane with the Turbo Trans-Blot system (Bio-Rad Laboratories, Lunteren, The Netherlands). The following primary antibodies were added to the membranes: mouse anti-MDM2 (IF2) (1:1000; Gibco; Thermo Fischer Scientific, Bleiswijk, The Netherlands; #33-7100), mouse anti-MDMX (1:1000; Merck KGaA, Darmstadt, Germany; #04-1555), anti-β-actin antibody (1:10,000; Thermo Fischer Scientific, Bleiswijk, The Netherlands; #MA5-15739), mouse anti-human p53 (1:1000; Dako Denmark A/S, Hilden, Germany; #M7001), rabbit anti-human p21 Waf1/Cip1 (1:1000; Cell Signaling Technology, Leiden, The Netherlands; #2947) and mouse anti-vinculin (1:4000; Merck KGaA, Darmstadt, Germany; #V9131), as a loading control. After incubating the membranes overnight at 4 °C, either goat anti-mouse IgG(H+L) cross-absorbed, HRP (1:2000; Invitrogen; Thermo Fischer Scientific, Bleiswijk, The Netherlands; #G-21040) or goat anti-rabbit IgG (H+L) cross-absorbed, HRP (1:2000; Invitrogen; #G-21234) were added as secondary antibodies and the membranes were incubated for 2 h at RT. To detect the proteins, Clarity Western ECL substrate (Bio-Rad Laboratories, Lunteren, The Netherlands) was added to the membranes.

### 4.5. CRISPR-Mediated Knock-Out Cell Lines

Both parental and resistant cells of NCCIT and 2102Ep were exposed to a ribonucleoprotein complex made of resuspension buffer R, 61 µM Cas9 protein and 100 µM gRNA duplex consisting of cRNA and tracrRNA, which was designed to target the gene of interest, *TP53* (Hs.Cas9.*TP53*.1.AA: CCATTGTTCAATATCGTTCCGGGG; Integrated DNA Technologies, Leuven, Belgium), using the Neon Transfection System (Thermo Fischer Scientific, Bleiswijk, The Netherlands). To confirm a successful *TP53* knock-out and to determine introduced mutations, of all the different cell lines, DNA was isolated using QuickExtract according to manufacturer’s protocol, followed by PCR with the following primers: forward: CAGTCAGATCCTAGCGTCGA and reverse: CACTGACAGGAAGCCAAAGG. Sequencing was performed by Macrogen Europe and knock-out efficiency was determined with the online ICE analysis tool by Synthego ICE analysis. Available online: https://ice.synthego.com/#/ (accessed on 15 September 2021).

### 4.6. Real-Time Quantitative Polymerase Chain Reaction (RT-qPCR)

#### 4.6.1. RNA Isolation

High-quality total RNA was extracted from the above-mentioned cell lines using TRIzol reagent (Life Technologies; Thermo Fischer Scientific, Bleiswijk, The Netherlands, cat.nr. 15596018) according to the manufacturer’s instructions. Quantity and quality were assessed on Nanodrop One (Isogen Lifescience B.V., de Meern, The Netherlands/Thermo Fischer Scientific, Bleiswijk, The Netherlands) and with Qubit 4 fluorometer (Invitrogen; Thermo Fischer Scientific, Bleiswijk, The Netherlands).

#### 4.6.2. miRNA Profiling

Targeted miRNA profiling was performed on diluted RNA (5 ng) using TaqMan MicroRNA Reverse Transcription Kit (Thermo Fischer Scientific, Bleiswijk, The Netherlands, cat.nr 4366597) and TaqMan Assays RNU48 (001006), hsa-miR371-3p (002124), and hsa-miR885-5p (002296) as described before [49]. RNU48 was used as for normalization and relative miRNA levels were computed as 2−ΔΔCt.

#### 4.6.3. mRNA Gene Expression

Diluted RNA (50 ng) was reverse transcribed using SuperScript IV reverse transcriptase (Thermo Fischer Scientific, Bleiswijk, The Netherlands, cat.nr. 18090050). RT-QPCR was run using the following TaqMan gene expression assays: *HPRT* (hs02800695_m1), *TP53* (hs01034249_m1), *TP73* (hs01056231_m1), *CDKN1A*/P21 (hs99999142_m1), *BBC3*/PUMA (hs00248075_m1), *PMAIP1*/NOXA (hs00560402_m1), *SOX2* (hs01053049_s1), *POU5F1* (hs00999632_g1), *POU5F1* (hs04195369_s1), *POU5F1* (hs03005111_g1). The 2× TaqMan Advanced PCR Master Mix (Thermo Fischer Scientific, Bleiswijk, The Netherlands, cat.nr. 4444556) was used and reactions were run in 96-well plates on QuantStudio 12K Flex System (Thermo Fischer Scientific, Bleiswijk, The Netherlands). HPRT was used as a housekeeping gene for normalization purposes and relative gene expression levels were computed as 2−ΔΔCt. Fold change between parental and knock-out clones was plotted.

### 4.7. Viability Assays

The sensitivity of the cells (both NCCIT and 2102Ep) to cisplatin was determined using a viability assay. To test the sensitivity of the cells to cisplatin, 10.000 NCCIT and 2102Ep parental cells and 4.000 2102Ep-resistant cells (based on performed seeding density assays; data not shown) were seeded in 100 µL RPMI or DMEM (respectively) medium per well in a 96-well plate. The plate was incubated at 37 °C and 5% CO_2_ overnight. Then, the following concentrations of cisplatin were made from 1 mg/mL cisplatin stock (Accord Healthcare B.V., Utrecht, The Netherlands) diluted in fresh medium enriched with 0.9% NaCl: 32 µM, 16 µM, 10 µM, 8 µM, 6 µM, 4 µM, 2 µM, 1 µM and 0.33 µM, or, 32 µM, 25 µM, 20 µM, 17.5 µM, 15 µM, 12.5 µM, 10 µM, 5 µM and 2.5 µM, dependent on expected IC50. The medium in the wells was replaced with 100 µL of the corresponding cisplatin medium. The plate was incubated at 37 °C and 5% CO_2_ for 72 h.

After incubation of either 2102Ep cells or NCCIT cells with cisplatin concentrations, CellTiter-Glo^®^ 2.0 Luminescent Cell Viability Assay kit (Promega, Leiden, The Netherlands) was used to lyse the cells to be able to measure the viability. Afterwards, 100 µL of the lysate was transferred to a Pierce white opaque 96-well plate (Thermo Fisher). The luminescence was measured on an ID3 Spectramax (Molecular Devices, San Jose, CA, USA) or a FLUOstar Omega (BMG Labtech, Ortenberg, Germany).

The viability assays were performed with three technical replicates and at least on three separate occasions (biological replicates). Data were visualized and interpreted using GraphPad Prism 9. To extract IC50 values, concentrations were transformed to logarithms, non-linear S-curves were fit through the data set using a GraphPad algorithm to extract absolute IC50s and the S-curves were interpolated with y = 0.5 to derive the absolute IC50s. Statistic differences were calculated using either an unpaired Student’s *t*-test or one-way ANOVA with a Tukey’s multiple comparisons post hoc test.

### 4.8. Genotyping with GSA Arrays

Genomic changes between original and knock-out subclones were identified after mono-clonal expansion and a minimum of ~3 months of separate cell culture using Infinium Global Screening Array-24 v3.0 BeadChipGSA (GSA) profiling. Array data were obtained from the HUGE-F as a Genome Studio vs. 2.0.4 (Illumina, Eindhoven, The Netherlands) project using the hg38 reference genome.

### 4.9. Data Visualization

Data were visualized using cBioPortal (v3.7.12), Adobe Illustrator (2020), SnapGene^®^ 5.3.2 and GraphPad Prism 9.

## 5. Conclusions

**Figure 5:**
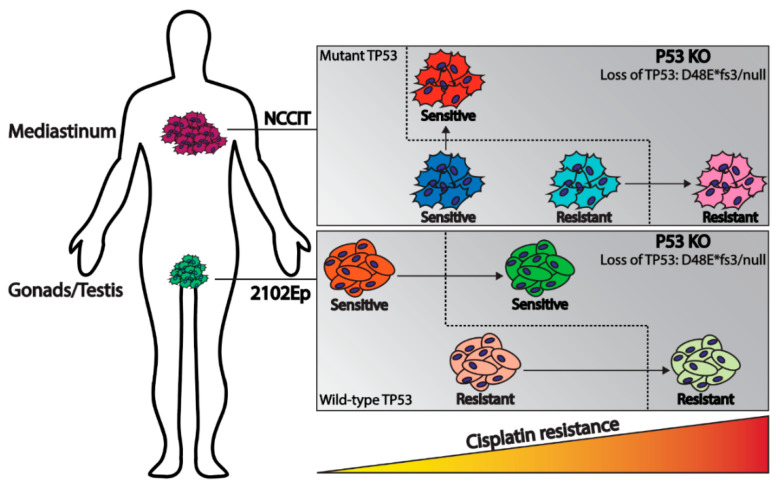
Schematic overview illustrating the model of this study. Mediastinal GCTs (NCCIT cell line) have a bias towards *TP53* mutations where testicular GCTs (2102Ep cell line) usually harbor wild-type *TP53*. In both cases knock-out of *TP53* results in increased cisplatin resistance.

In conclusion, this study, combining GCT patient data characteristics and functional mechanistic cell line investigations, illustrates the role of *TP53* status in cisplatin resistance in GCTs, related to the anatomical location associated with molecular constitution. The results obtained show that the investigated cell lines, independent of intrinsic resistance, demonstrate a beneficial effect of the loss of *TP53* regarding cisplatin resistance, as schematically represented in Figure 5. Furthermore, it is interesting to note that the hemizygous mutant *TP53*, originally present in the commonly used NCCIT, is functional in the context of cisplatin sensitivity, as the knock-out of this mutant resulted in increased cisplatin resistance. The isogenic generated cell lines provide a novel informative model system to study the involvement of *TP53* in the original cellular background (of NCCIT ad 2102Ep), and provide insight into the clinical behavior of GCTs. Moreover, we provide, to our knowledge, for the first time, insights into the functionality of the hemizygous *TP53* mutant present in NCCIT, and, additionally, we developed a cell line harboring a bona fide *TP53* null status. More GCT cell lines originating from both tumor sites (i.e., mediastinum and testis), or even tumor-derived organoids from these sites, could provide more insights into the role of *TP53* in the clinical behavior and chemotherapy response of these tumors. These data can aid in patient stratification for optimal clinical decision making, especially for mediastinal tumors in which *TP53* mutations are more common. Patients could benefit from screening for intrinsic *TP53* mutations in the primary tumor or acquired *TP53* mutations in the refractory malignancies. This study illustrates the contribution of *TP53* not only to known cisplatin sensitivity, but also as a potential target for acquired cisplatin resistance.

## Data Availability

The data presented in this study are available on request from the corresponding author.

## Supplementary Materials

The following are available online at www.mdpi.com/article/10.3390/ijms222111774/s1.
