# Supplementary material for: The Role of TP53 in Cisplatin Resistance in Mediastinal and Testicular Germ Cell Tumors
Source: Int J Mol Sci. 2021 Oct 29;22(21):11774. doi: 10.3390/ijms222111774 (PMC8583723; doi:10.3390/ijms222111774)
Supplement: Supplementary file 1 [file ijms-22-11774-s001.zip › ijms-1422137-supplementary.pdf]

International Journal of
Molecular Sciences

MDPI

Article

The role of *TP53* in cisplatin resistance in mediastinal and testicular germ cell tumors.

Dennis M. Timmerman, Thomas F. Eleveld, Ad J. M. Gillis, Carlijn C. Friedrichs, Sanne Hillenius, Tessa L. Remmers, Sruthi Sriram, and Leendert H. J. Looijenga*

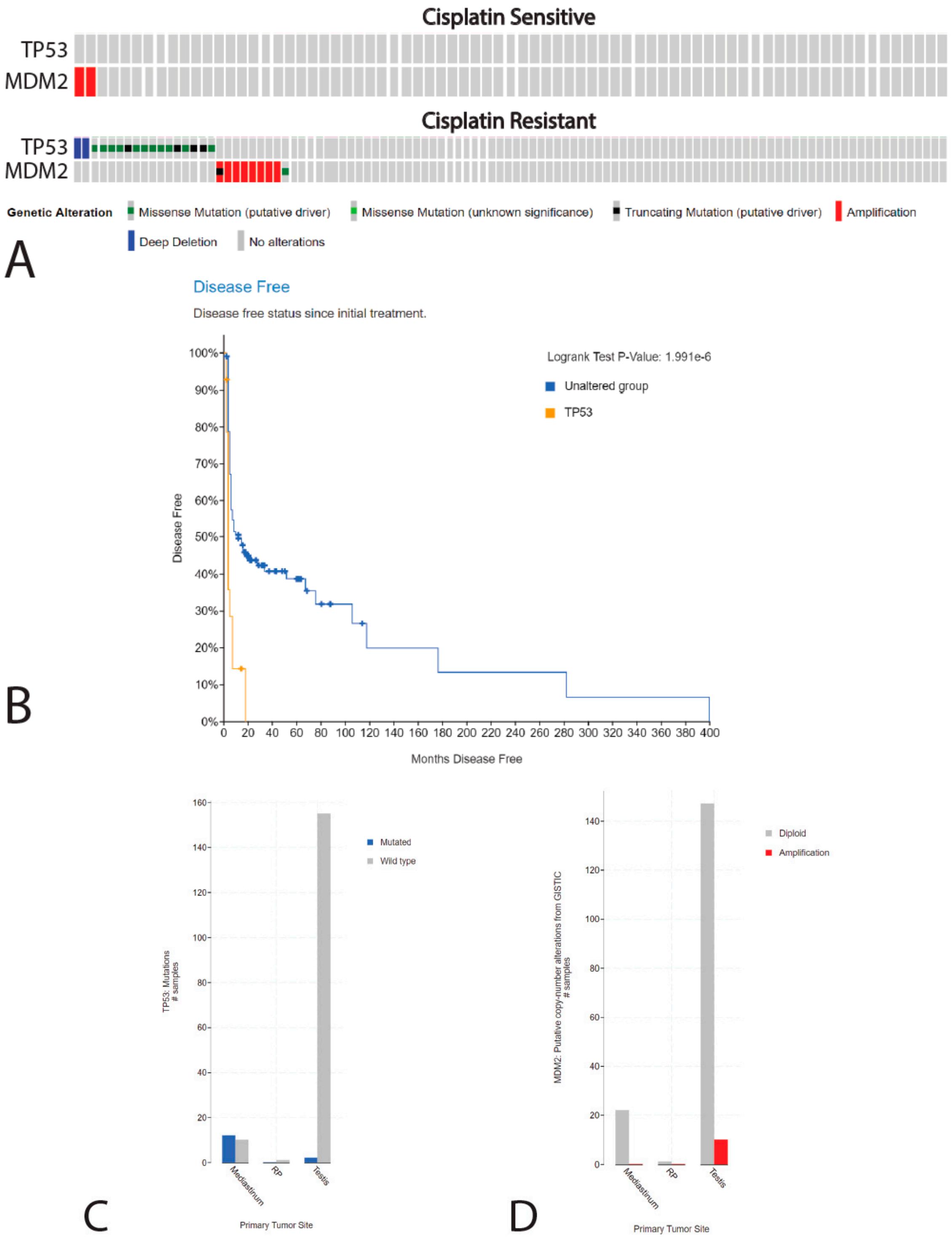

Figure S1. cBioPortal analysis of the MSKCC, J Clin Oncol 2016 data set. (A) Oncoprint of refractory GCT patients classed as either "cisplatin sensitive" or "cisplatin resistant". Displayed are the genomic alterations for *TP53* and *MDM2*. (B) Disease-free survival plot of patients harboring no alterations in *TP53* (Unaltered group) or patients that do harbor alterations in *TP53* (*TP53*). A significant reduction in disease-free survival can be observed in patients harboring *TP53* alterations (Logrank Test P-value: 1.99e-6). (C) Bar graph displaying the primary tumor site of patients with wild type (grey) or mutated (blue) *TP53*. (D) Bar graph displaying the primary tumor site of patients with wild type (grey) or amplified (red) *MDM2*. (9),.

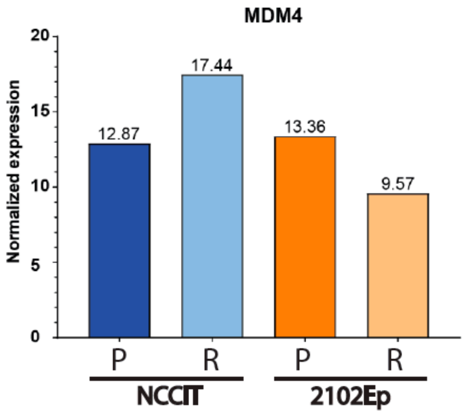

Figure S2. Normalized expression of *MDM4* (RNAseq) in NCCIT and 2102Ep (both parental and resistant).

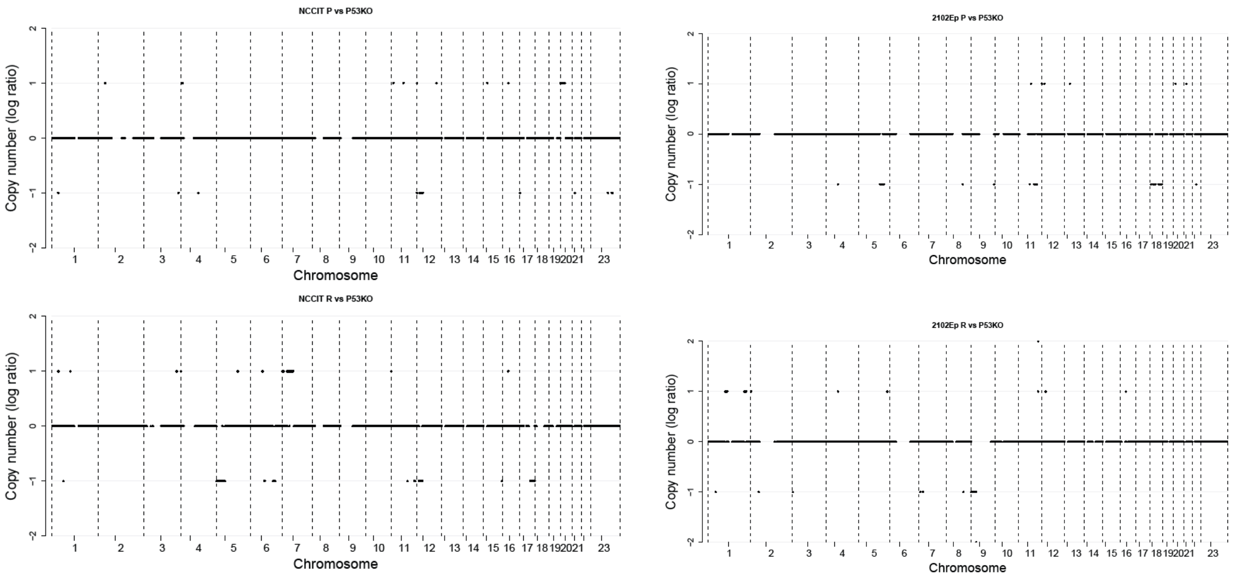

Figure S3. Differential GSA copy number analysis of original and matched knock-out cell lines. No major differences could be observed.
